# Terminally Differentiated Epithelial Cells of the Thymic Medulla and Skin Express Nicotinic Acetylcholine Receptor Subunit ***α***3

**DOI:** 10.1155/2014/757502

**Published:** 2014-07-03

**Authors:** Aichurek Soultanova, Alexandra R. Panneck, Amir Rafiq, Wolfgang Kummer

**Affiliations:** Institute of Anatomy and Cell Biology, German Center for Lung Research, Justus-Liebig-University Giessen, Aulweg 123, 35385 Giessen, Germany

## Abstract

In the thymus, T cell maturation is influenced by cholinergic signaling, and the predominantly expressed receptor is the *α*3-subunit of nicotinic acetylcholine receptors, encoded by the *chrna3* gene. We here determined its cellular distribution utilizing an appropriate eGFP-expressing reporter mouse strain. Neither T cells (CD4, CD8) nor mesenchymal cells (desmin-positive) expressed eGFP. In the thymic medulla, eGFP-positive cells either were scattered or, more frequently, formed small clusters resembling Hassall's corpuscles. Immunolabeling revealed that these cells were indeed terminally differentiated epithelial cells expressing keratin 10 (K10) but neither typical cortical (K8, K18) nor medullary keratins (K5, K14). These labeling patterns reflected those in the epidermis of the skin, where overlap of K10 and eGFP expression was seen in the stratum granulosum, whereas underlying basal cells displayed K5-immunoreactivity. A substantial portion of thymic eGFP-positive cells was also immunoreactive to chromogranin A, a peptide previously reported in epidermal keratinocytes in the stratum granulosum. Its fragment catestatin has multiple biological activities, including suppression of proinflammatory cytokine release from macrophages and inhibition of *α*3*β*4 nAChR. The present findings suggest that its thymic production and/or release are under cholinergic control involving nAChR containing the *α*3-subunit.

## 1. Introduction

The thymus is the site of step-wise maturation of naïve T cells from immature thymocytes which occurs along with positive and negative selection processes while thymocytes migrate from the cortex to the medulla. These processes are influenced by cholinergic signaling [1–4], and acetylcholine (ACh) is endogenously synthesized in the thymus [5–7].

Signaling via nicotinic ACh receptors (nAChR) has received particular attention. These receptors are pentamers composed of various subunit combinations. The “muscle type” nAChR originally identified at the motor endplate consists of two *α*1-, one *β*1-, one *ε*- (or *γ*- at fetal stage), and one *δ*-subunit, and these are also expressed by myoid [8, 9] and epithelial cells of the thymic medulla [10–13]. Thymic expression and presentation of muscle-type nAChR subunits have been associated with a frequent (85%) variant of myasthenia gravis, a disease of the motor endplate, where autoantibodies against such subunits are formed, as the thymus frequently shows abnormal structure in this condition and thymectomy is beneficial for the patients [14].

“Neuronal” nAChR are homo- or heteromers of *α*-subunits 2–7 and 9-10 (*α*8 is expressed only in chicken) and *β*-subunits 2–4 [15–17]. Despite their designation as “neuronal” they are widely expressed outside the nervous system including the thymus [18, 19]. Amongst them, subunits *α*3, *α*5, and *β*4 exhibit highest expression in early postnatal mouse thymus, reaching 7–15% of mRNA content found in brain as standard [19]. Their genes—*chrna3*,* chrna5,* and* chrnb4*—are clustered on chromosome 9 in mice, and, when coexpressed, the translated proteins assemble to functional *α*3(*α*5)*β*4 nAChR with either 2 *α*- and 3 *β*-subunit or 3 *α*- and 2 *β*-subunit chains [15, 20]. The *α*3-subunit is essential for receptor function and may occur with or without an additional *α*5-subunit in these receptors while the *α*5-subunit does not form functional receptors without additional *α*-subunits [15]. Messenger RNAs coding for these subunits have been detected in isolated thymocytes and cultured thymic epithelial cells (TEC) obtained from children undergoing corrective cardiac surgery [18], but it is still unclear which specific thymic cell types express these receptors* in situ*.

In view of the reported specificity problems associated with immunohistochemical detection of nAChR subunits [21], we utilized a reporter mouse strain expressing eGFP under the control of the* chrna3 *promoter coding for the essential *α*3-subunit [22]. To further characterize and identify eGFP-positive cells in the thymus, tissue sections were subjected to immunohistochemistry with marker antibodies for subsets of thymocytes (CD4, CD8), myoid and mesenchymal (desmin) and thymic epithelial cells. Thymic epithelial cells are heterogeneous and can be classified according to various criteria. A broad classification divides them into four general types (with further subpopulations [23, 24]): (a) subcapsular/paraseptal/perivascular, (b) cortical, (c) medullary (mTEC), and (d) terminally differentiated mTEC, usually arranged in Hassall's corpuscles [25–28]. These types differ in their intermediate filament content, with cortical and a small population of mTEC expressing keratins typical for simple epithelia (K8, K18) [23, 28, 29], the majority of mTEC keratins characteristic for immature basal cells of stratified epithelia, that is, K5 and K14 [24, 28, 30], and terminally differentiated cells of Hassall's corpuscles (in human) and Hassall's corpuscles-like structures (in mice) expressing K10 [31].

## 2. Materials and Methods

### 2.1. Animals and Tissue Collection

Transgenic mice expressing eGFP under* chrna3 *promoter [22] were killed with an overdose of isoflurane (Abbott, Wiesbaden, Germany). Animals of either gender (*N* = 9) were transcardially perfused with heparin-containing rinsing solution [32] followed by either 4% paraformaldehyde in 0.1 M phosphate buffer (pH = 7.4) or Zamboni fixative (2% paraformaldehyde, 15% saturated picric acid in 0.1 M phosphate buffer, pH 7.4). Thymi and hairy skin of the head were dissected and fixed overnight by immersion in the same fixative used for perfusion. In 5 additional animals, tissues were immersion-fixed overnight in Zamboni fixative without prior perfusion. Specimens were washed in 0.1 M phosphate buffer, pH = 7.4, for 30 h, incubated overnight in 18% sucrose in 0.1 M phosphate buffer, and frozen in OCT compound (Sakura Finetek, Staufen, Germany) using liquid nitrogen.

### 2.2. Immunohistochemistry

Thymi and hairy skin of the head were cut with a cryostat into 4–10 *μ*m thick sections and air-dried for 1 h. Nonspecific protein binding sites were saturated with 10% horse serum, 0.5% Tween, and 0.1% BSA in PBS (0.005 M phosphate buffer, pH = 7.4, with 0.45% NaCl). Sections were incubated for 16 h with primary antibodies diluted in PBS with doubled salt concentration and containing 0.01% NaN_3_. Each antibody was applied to thymi from at least 6 animals. Antibody dilutions and sources are specified in Table 1. After washing in PBS, sections were covered with Cy3-conjugated donkey anti-rabbit IgG (1 : 2000; Chemicon, Darmstadt, Germany) or donkey anti-rat IgG (1 : 1000; Dianova, Hamburg, Germany) for 1 h, washed, postfixed in 4% paraformaldehyde in 0.1 M phosphate buffer, washed again, and mounted in carbonate-buffered glycerol (1 : 1, pH = 8.6). Controls were run by replacing primary antibodies with unrelated isotypes from the same species and by omission of first antibodies. Sections were evaluated with an Axioplan 2 epifluorescence microscope equipped with an AxioCam MRm camera system (Zeiss, Jena, Germany). Except overall adjustment of brightness, no further manipulations of digital images were performed.

## 3. Results

**Figure 1:**
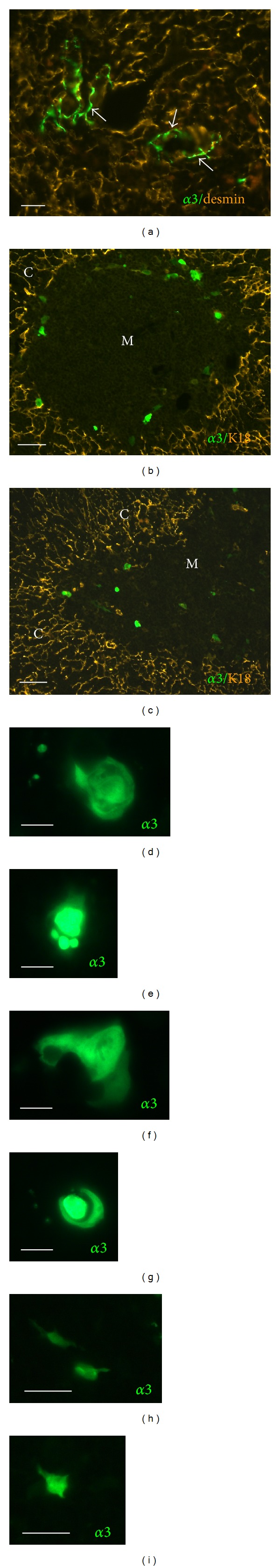
*Chrna3*-driven eGFP expression in the thymic medulla. (a) Perivascular nerve fibers (*arrows*) are *α*3-subunit-positive. Mesenchymal elements labeled with desmin antibody for better orientation (scale bar, 20 *μ*m). (b), (c) *α*3-subunit-expressing cells are located in the thymic medulla and at the corticomedullary junction. Cortex delineated by immunolabeling with K18-antibodies (scale bar, 50 *μ*m). (d)–(g) eGFP-Positive cells either form Hassall's corpuscles-like aggregates (scale bar, 10 *μ*m) or lie singularly (h), (i) (scale bar, 20 *μ*m). M: medulla; C: cortex.

**Figure 2:**
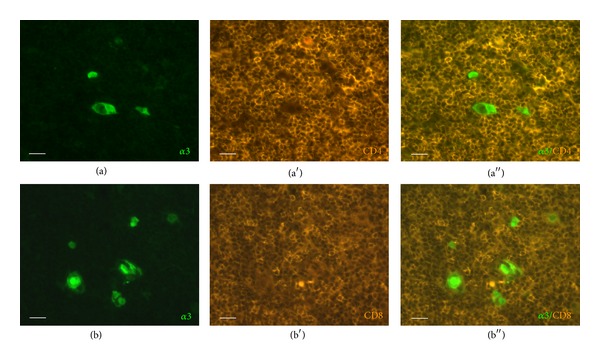
eGFP-positive cells are not thymocytes. Immunostaining for CD4 (a)–(a′′) and CD8 (b)–(b′′) revealed no colocalization with eGFP signal. Scale bar, 20 *μ*m.

The major nicotinic receptor mediating synaptic transmission in autonomic ganglia is a heteropentamer of *α*3(*α*5)*β*4 subunits [15, 33]. Accordingly, autonomic nerve fibers surrounding thymic arteries exhibited intense eGFP fluorescence (Figure 1(a)). Besides these nerve fibers, positive cells were observed in the thymic medulla with some preference to the corticomedullary junction, but practically sparing the external cortex (Figures 1(b) and 1(c)). Such cells typically formed clusters. The larger clusters showed characteristic morphology of murine Hassall's corpuscle-like structures. Infrequently observed singular eGFP-positive cells and those forming groups of two or three, displayed variable shape, ranging from round to oval to elongated with processes emerging from the cell body (Figures 1(d)–1(i)). According to this complex shape, these eGFP-positive cells were not thymocytes and did not express CD4 or CD8 (Figure 2).

**Figure 3:**
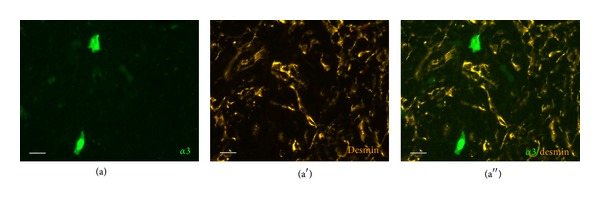
Alpha3-subunit-expressing cells are neither myoid nor mesenchymal cells, since there is no colocalization with desmin-immunoreactivity (scale bar, 20 *μ*m).

Desmin is a marker for thymic myoid cells and mesenchymal cells [9]. Accordingly, immunolabeling revealed a network of fine processes in the thymic medulla and a few positive cells bodies. Colocalization with eGFP fluorescence was not observed (Figure 3).

**Figure 4:**
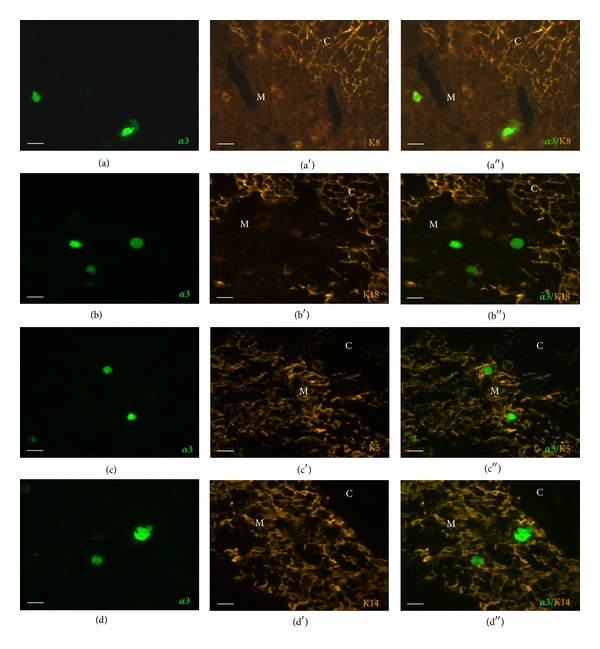
Neither cortex- (K8, K18) nor medulla-specific (K5, K15) cytokeratins are expressed by *α*3-subunit-positive cells. Immunostaining with K8 (a)–(a′′), K18 (b)–(b′′), K5 (c)–(c′′), and K14 (d)–(d′′) antibodies. Scale bar, 20 *μ*m.

**Figure 5:**
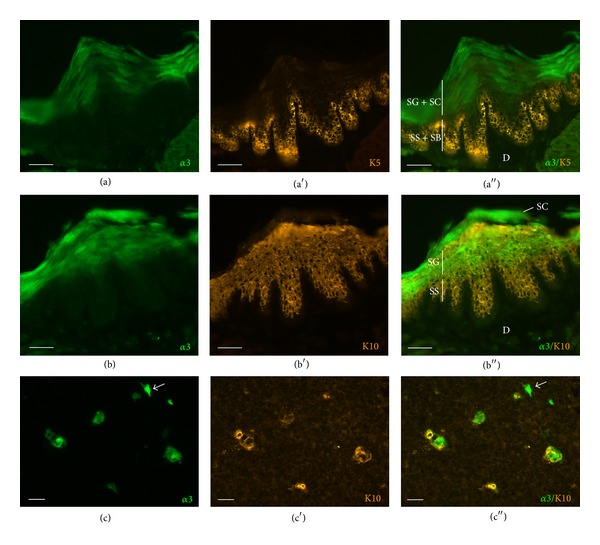
Alpha3-subunit-positive cells are terminally differentiated epithelial cells. (a)–(a′′)* Chrna3*-driven eGFP fluorescence in the stratum granulosum (SG) and stratum corneum (SC) of the epidermis, K5-immunoreactivity in the lowermost stratum spinosum (SS) and stratum basale (SB). D: dermis. Scale bar, 50 *μ*m. (b)–(b′′) K10-immunoreactivity in stratum granulosum and stratum spinosum, overlapping with eGFP signal in stratum granulosum. Scale bar, 50 *μ*m. (c)–(c′′) The majority of *α*3-subunit-expressing cells in the thymus are K10-immunoreactive;* arrow* points to an infrequently occurring K10-negative cell. Scale bar, 20 *μ*m.

K8- and K18-immunoreactive epithelial cell processes formed a dense network in the cortex, and a small population of less branched mTEC was also K8- and K18-immunoreactive, as previously described [34]. Neither of these antibodies, however, labeled eGFP-positive cells (Figures 4(a) and 4(b)). In the medulla, a dense mesh of K5- and K14-immunoreactive cell processes was noted. Such processes surrounded eGFP-positive cells which were larger than keratin-immunoreactive cell bodies and were K5/K14-negative (Figures 4(c) and 4(d)). This spatial arrangement resembled that of the epidermis where K5 is expressed by the basal cells and its expression ceases when keratinocytes terminally differentiate in the stratum granulosum [35, 36]. Accordingly, we observed eGFP fluorescence in the K5-negative stratum granulosum of hairy skin and K5-positive cells in the underlying stratum basale and the lowermost stratum spinosum (Figure 5(a)). In the epidermis, keratinocytes switch to K10 expression in the stratum spinosum which is kept in the stratum granulosum. This partly overlaps with eGFP fluorescence which is first seen in the stratum granulosum and extends into the stratum corneum (Figure 5(b)). The same pattern of extensive but not complete overlap of eGFP- and K10-immunoreactivity was found in the thymus (Figure 5(c)).

**Figure 6:**
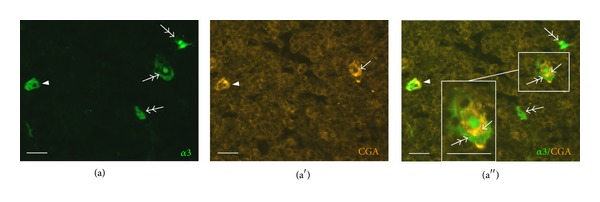
Immunolabeling for CGA. Both double- (eGFP^+^/CGA^+^,* arrowhead*) and single-labeled cells (eGFP^+^/CGA^−^,* double-headed arrow*; eGFP^−^/CGA^+^,* arrow*) can be observed. CGA-immunoreactive and eGFP-positive cells occur together in Hassall's corpuscle-like structures (higher magnification in inset). Scale bar, 20 *μ*m.

In addition, there was partial colocalization of eGFP with chromogranin A (CGA) in the thymus. CGA-immunoreactive cells were found mainly in the medulla, often attached to Hassall's corpuscle-like structures, as previously reported [37, 38] (Figure 6). CGA-immunoreactive granules were observed in a population of eGFP-positive cells, although CGA^+^/eGFP^−^ and CGA^−^/eGFP^+^ cells were also present in about equal proportions (Figure 6).

## 4. Discussion

The present study demonstrates that the most abundantly expressed nAChR subunit in the murine thymus, the *α*3-subunit, is localized to a distinct mTEC type, that is, terminally differentiated mTEC expressing K10. In the skin, eGFP expression driven by the* chrna3 *promoter was more restricted than expected from previous immunohistochemical studies utilizing *α*3-subunit antibodies [39, 40], which possibly originates from the noted specificity problems of nAChR subunit antibodies documented by the use of respective gene-deficient mice [21]. In the epidermis, where keratinocytes undergo stepwise differentiation while migrating through the different layers towards the surface, we saw K10 prior to eGFP expression and eGFP shortly after keratinization when K10 was no longer detectable. This sequence of expression (K10 before nAChR*α*3) suggests that the known promoting effect of nicotine on K10 expression by keratinocytes [41] is not driven by *α*3-subunit containing nAChR but likely by another nAChR subtype, for example, *α*7 or *α*9*α*10 nAChR, also known to be expressed in keratinocytes [39, 41–43].

Assuming the same sequence of differentiation in Hassall's corpuscle-like structures, the few K10^+^/eGFP^−^ and K10^−^/eGFP^+^ cells in the thymic medulla would not represent an entirely distinct cell population but mTEC at corresponding intermediate stages of differentiation. When K10 is ectopically expressed in K5-positive mTEC, the thymus presents premature involution with increased apoptosis and reduced proliferation of thymocytes [44] but this does not necessarily allow conclusions about physiologically K10-expressing terminal mTEC.

Terminally differentiated mTEC, also characterized by expression of involucrin, no longer express the autoimmune regulator (Aire) which plays a pivotal role in establishing self-tolerance by negative selection and FoxP3^+^ regulatory T cell (Treg) production [31]. Human Hassall's corpuscles express thymic stromal lymphopoietin which activates medullary dendritic cells to express high levels of CD28 ligands (CD80 and CD86) [45]. Such activated dendritic cells induce the generation of CD4^+^CD8^−^CD25^+^ T cells, leading to the suggestion that Hassall's corpuscles, via dendritic cells, trigger secondary positive selection of medium-to-high affinity self-reactive T cells resulting in Treg generation in the thymic medulla [45]. In mice, however, Treg develop normally in lymphotoxin *β*-receptor-deficient mice which lack terminally differentiated mTEC as judged from the absence of involucrin^+^ cells in the medulla [31, 46]. Thus, the function of terminally differentiated mTEC clusters is still unclear.

CGA, originally isolated from chromaffin cells of the adrenal medulla and later found to be produced by other endocrine cell types, is also expressed in epidermal keratinocytes in the stratum granulosum [47] which is again in parallel to CGA-immunoreactivity in Hassall's corpuscles [37, 38]. Its peptide fragment catestatin has multiple biological activities, including suppression of proinflammatory cytokine release by and increasing p-STAT3 levels in peritoneal and bone marrow-derived macrophages [48]. The present findings suggest that its thymic production and/or release are under cholinergic control involving nAChR containing the *α*3-subunit. On the other hand, catestatin is an inhibitor of *α*3*β*4 nAChR [49], so that it might act in an autoinhibitory feedback in *α*3-subunit-expressing terminally differentiated mTEC.

Except terminally differentiated mTEC and autonomic nerve endings, no further cellular elements of the thymus expressed* chrna3*-driven eGFP in the present study. In contrast, RT-PCR revealed mRNA coding for *α*3- and *β*4-subunits in isolated human CD4^+^CD8^+^ thymocytes [18]. This discrepancy might be due to species differences or incomplete expression of the transgene in the mouse strain used in this study.

In conclusion, the present study identifies nAChR *α*3-subunit expression by terminally differentiated mTEC. Their function is still unclear, which has been ascribed at least partly to the lack of adequate models allowing for isolating these cells [31], a problem that might be overcome utilizing the nAChR*α*3^BAC^-eGFP mouse strain analyzed in this study.

## Figures and Tables

**Table 1:** Primary antibodies used in the study.

Target	Immunogen	Host	Clone	Dilution	Catalog number	Company
CD4	Not specified	Rat	Monoclonal, clone RM4-5	1 : 400	IH93-0042-91	eBioscience, Frankfurt am Main, Germany

CD8	Not specified	Rat	Monoclonal, clone 53-6.7	1 : 400	IH93-0081-91	eBioscience, Frankfurt am Main, Germany

Chromogranin A	Synthetic peptide corresponding to residues near the C-terminus of human chromogranin A	Rabbit	Polyclonal	1 : 25	1782-1	Epitomics, Cambridge, UK

Desmin	Synthetic peptide corresponding to C-terminus of human desmin	Rabbit	Monoclonal, clone Y66	1 : 200–1 : 800	04-585	Merck Millipore, Darmstadt, Germany

Keratin 5	Synthetic peptide corresponding to C-terminus of human keratin 5	Rabbit	Monoclonal, clone SP27	1 : 50–1 : 200	SPB-M3270	Spring, Pleasanton, CA, USA

Keratin 8	Synthetic peptide corresponding to C-terminus of human keratin 8	Rabbit	Monoclonal, clone SP102	1 : 50	SPB-M4020	Spring, Pleasanton, CA, USA

Keratin 10	C-terminus of the mouse keratin 10	Rabbit	Polyclonal	1 : 400	PRB-159P	Covance, Münster, Germany

Keratin 14	Synthetic peptide corresponding to C-terminus of human keratin 14	Rabbit	Monoclonal, clone SP53	1 : 400	SPB-M3534	Spring, Pleasanton, CA, USA

Keratin 18	Synthetic peptide corresponding to C-terminus of human keratin 18	Rabbit	Monoclonal, clone SP69	1 : 200	SPB-M3694	Spring, Pleasanton, CA, USA
